# Distribution of H3K27me3, H3K9me3, and H3K4me3 along autophagy-related genes highly expressed in starved zebrafish myotubes

**DOI:** 10.1242/bio.029090

**Published:** 2017-10-12

**Authors:** Peggy R. Biga, Mary N. Latimer, Jacob Michael Froehlich, Jean-Charles Gabillard, Iban Seiliez

**Affiliations:** 1 Department of Biology, University of Alabama at Birmingham, Birmingham, AL 35294, USA; 2 INRA, UR1037 Laboratory of Fish Physiology and Genomics, Campus de Beaulieu, F-35042 Rennes, France; 3 INRA-UPPA, UMR1419 Nutrition Metabolisme Aquaculture, F-64310 St-Pée-sur-Nivelle, France

**Keywords:** Zebrafish, Histone modification, Atrophy, Autophagy, Myotube, Epigenetic

## Abstract

The zebrafish (*Danio rerio*) remains the teleost fish of choice for biological investigations due to the vast array of molecular tools and resources available. To better understand the epigenetic regulation of autophagy, we utilized a primary myotube culture system generated from isolated myogenic precursor cells (MPCs) from zebrafish grown under starvation conditions using a media devoid of serum and amino acids. Here, we report starvation-induced regulation of several autophagy-related genes (*atg*) expression and profile the distribution of H3K27me3, H3K9me3, and H3K4me3 marks along *lc3b*, *atg4b* and *p62/sqstm1* loci. These data support epigenetic regulation of autophagy in response to starvation that suggests a level of regulation that can be sustained for chronic conditions via chromatin modification.

## Abstract (precis)

**Summary:** The role of nuclear events in the control of autophagy is underappreciated. This study demonstrates cell starvation associated gene expression changes and autophagy-specific histone modifications.

## INTRODUCTION

In vertebrates, starvation induces muscle wasting by decreasing protein synthesis while increasing protein degradation, which is regulated via highly conserved proteolytic pathways (Hershko et al., 2000) including the ubiquitin/proteosomal pathway (UPS) and macroautophagy (hereafter referred to as autophagy) (Sandri, 2010). Autophagy aids in maintaining cellular homeostasis by degrading cytoplasmic components and recycling long-lived proteins, while the UPS pathway degrades transient short-lived proteins (Ciechanover, 2005). Together, these two processes degrade proteins into smaller peptides and aid in maintaining the amino acid pools and energy balance during acute or long-term starvation (see reviews from Levine and Yuan, 2005; Glickman and Ciechanover, 2002; Baehrecke, 2005).

Upon starvation or other stress conditions, autophagy is induced by both a fast transcription-independent mechanism, entirely mediated by post-translational protein modifications, and a slower transcription-dependent mechanism involving a complex regulatory network (Feng et al., 2015). Most research has focused on the short-term post-translational modifications of autophagy-related proteins, and the underlying mechanisms are now well described (Yin et al., 2016). By comparison, we know much less about the long-term transcriptional regulation of autophagy. Only recently have researchers started to identify an entire network of transcription factors that are involved in the long-term outcome of autophagy (Füllgrabe et al., 2016; Shin et al., 2016a). An increasing number of transcription factors have thus been linked to the transcriptional activation of autophagy-related genes involved in all steps of the process (Füllgrabe et al., 2016).

However, it has become more than evident that the nuclear impact on autophagy is not limited to the sole action of transcription factors, but also involves epigenetic mechanisms (Füllgrabe et al., 2014a; Baek and Kim, 2017). Histone modifications exerted by CARM1 H3R17 methyltransferase (Shin et al., 2016b), G9a H3K9 methyltransferase (Artal-Martinez de Narvajas et al., 2013), EZH2 H3K27 methyltransferase (Wei et al., 2015), SIRT1 H4K16 deacetylase, and its counterpart hMOF H4K16 acetyltransferase (Füllgrabe et al., 2013), have been reported as critical nuclear events of autophagy. However, the molecular basis for this epigenetic regulation of autophagy remains poorly understood.

In this context, the present study used the recently validated *in vitro* myogenic culture system from zebrafish (Froehlich et al., 2014, 2013) to profile the distribution of H3K27me3, H3K9me3 and H3K4me3 histone methylation marks during *in vitro* starvation along loci displaying key roles in the control of autophagy.

## RESULTS AND DISCUSSION

**Fig. 1.**
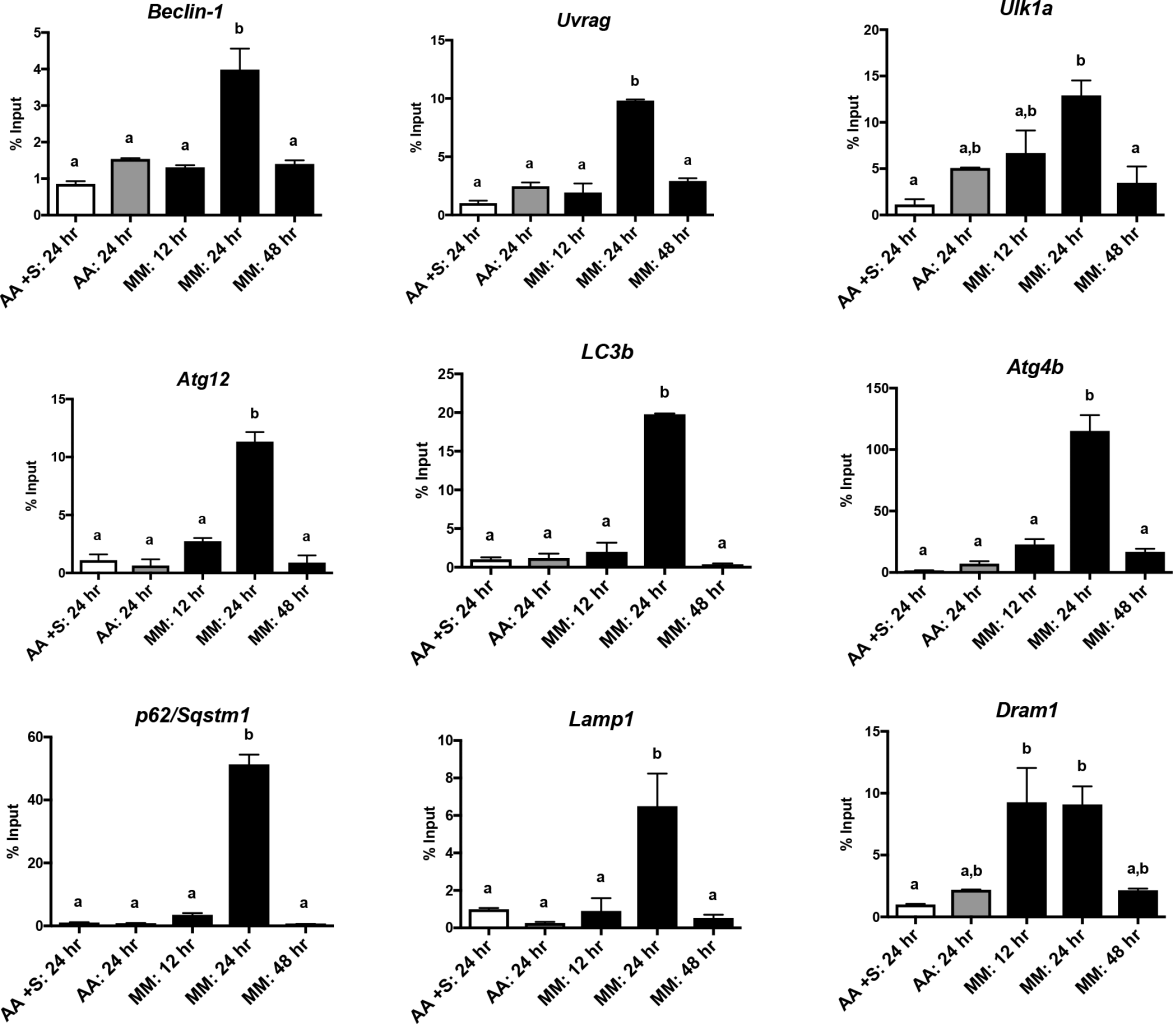
**Cell Starvation upregulates genes associated with autophagy.** Quantification of gene expression levels in zebrafish myotubes, *in vitro*, following incubation in normal media with amino acids and serum (AA+S; white bars), normal media with amino acids and no serum (AA; gray bars), or minimum media lacking amino acids and serum (MM; black bars). Genes analyzed include: *Beclin-1*; *Uvrag*; *Ulka1*; *Atg12*; *LC3b*; *Atg4b*; *p62/Sqstm1*; *Lamp1*; and *Dram1*. Relative gene expression data are represented as ΔΔCt values corrected for *beta-actin* levels (*n*=3 pools; different letters represent significance at *P*<0.05, ANOVA, Tukey's multiple comparisons). Error bars indicate s.e.m.

The maintenance of skeletal muscle mass depends on the balance between protein synthesis and degradation. A synthesis:degradation ratio of >1 results in maintenance and/or hypertrophy of muscle mass, while a ratio of <1 leads to muscle wasting (i.e. atrophy, cachexia, sarcopenia). Protein degradation occurs in part through the mechanisms of autophagy, a process orchestrated by autophagosome generation. As expected, the incubation of *de novo* zebrafish myotubes generated from isolated myogenic precursor cells (MPCs) in minimal media (devoid of serum and amino acids) resulted in the upregulation of several genes known to function in the autophagic pathway (Fig. 1). Such an induction of autophagy-related genes in restrictive media is now well established, and would allow a sustained level of autophagy flux in the cells (Füllgrabe et al., 2014b; Shin et al., 2016b). To date, more than 20 transcription factors have been identified to be linked to the autophagic process (Pietrocola et al., 2013; Füllgrabe et al., 2016); however, their interactions and respective roles remain far from being understood. Here we demonstrate that *de novo* zebrafish myotubes generated from isolated MPCs constitute a useful model to gain insights on these complex mechanisms involved in the transcriptional regulation of autophagy.

**Fig. 2.**
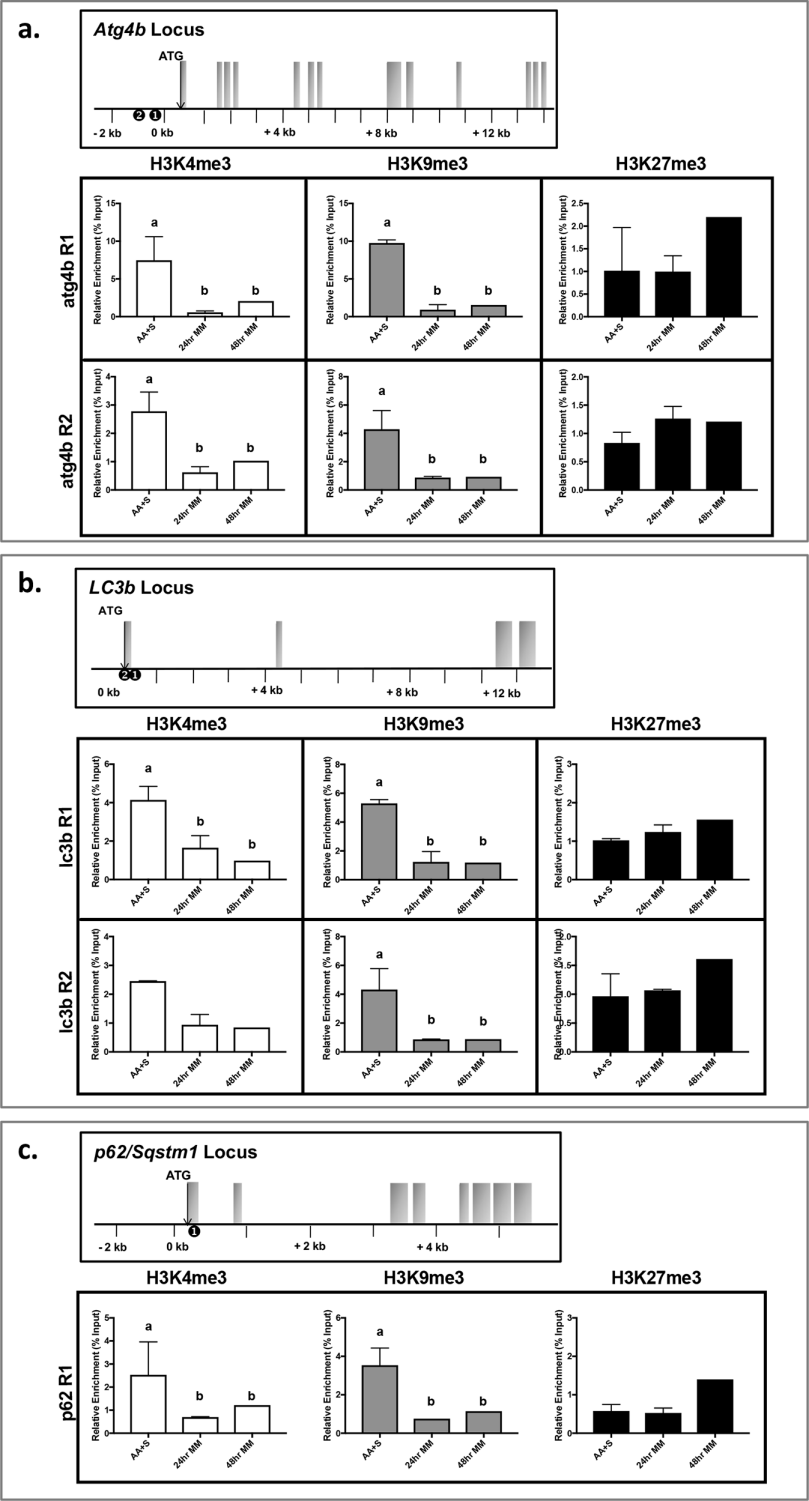
**Chromatin immunoprecipitation (ChIP) and subsequent quantitative PCR were utilized to identify gene-specific region association with trimethylation histone 3 lysine residues (H3K4, H3K9, and H3K27).** Regions of association are shown for each gene locus (➊ and ➋) above respective qPCR data for (A) *Atg4b*; (B) *LC3b*; and (C) *p62/Sqstm1*. In each gene locus diagram, exons are shown as shaded boxes with the ATG start site shown with a down-facing arrow. Gene expression data are represented as ΔΔCt values corrected for percent input. Values are means±s.e.m., *n*=3, mean of two replications. Different letters represent significantly different values (*P*<0.05, Student's *t*-test).

In recent years, compelling evidence has revealed the important role of epigenetic processes in the control of autophagy (Füllgrabe et al., 2014b; Baek and Kim, 2017). The transcriptional state of several autophagy-related genes has thus been shown to be under the strict control of epigenetic imprinting through histone methylation (Shin et al., 2016b; Artal-Martinez de Narvajas et al., 2013; Wei et al., 2015). In the present study, we therefore sought to profile the distribution of H3K27me3, H3K9me3, and H3K4me3 modifications along three autophagy-related genes (*atg4b*, *p62/sqstm1* and *lc3b*) highly expressed in starved zebrafish myotubes (Fig. 1). The obtained results show that all three genes exhibited high H3K4me3 and H3K9me3 levels at the studied regions in cells cultivated in the control medium (AA+S) (Fig. 2). In contrast, no H3K27me3 enrichment has been recorded in the control cells compared to those grown in the minimal medium.

Enrichment in H3K4me3 and H3K9me3 was reduced at both 24 and 48 h of starvation in *Atg4b* locus (regions 1 and 2, *P*<0.001) and *p62/Sqstm1* locus (region 1, *P*=0.0176) with reductions correlating with the increased transcription of both *Atg4b* and *p62/sqstm1* at 24 h. Similarly, across the *LC3b* locus, region 2 exhibited reduced enrichment (*P*=0.016) of H3K9me3 and region 1 exhibited reduced enrichment of both H3K4me3 and H3K9me3 (*P*=0.0001) during starvation. These chromatin modification changes correspond with increased *LC3b* transcription upregulation at 24 h post starvation. The increased transcription of *Atg4b*, *LC3b*, and *p62/Sqstm1* 24 h following starvation, along with the reduction of the repressive H3K9me3 mark, suggests some degree of epigenetic regulation of these genes in starved zebrafish myotubes. To our knowledge, no data have been reported before on the enrichment in H3K9me3 at the autophagy-related loci. However, a recent study showed that H3K9 dimethylation (H3K9me2) mediated by the histone methyltransferase G9a acts as a repressor of autophagy (Artal-Martinez de Narvajas et al., 2013). Under normal conditions, G9a associates with the promoter of autophagy-related genes *lc3b*, *wipi1*, and *dor*, epigenetically repressing them; however, G9a and G9a-repressive histone marks are removed during starvation. Interestingly, although the G9a has been most associated to the generation of H3K9me1 and H3K9me2, it may also instigate tri-methylation either directly (Osipovich et al., 2004; Yokochi et al., 2009) or through the recruitment or activation of other histone methylases (Freitag and Selker, 2005). In this regard, in the absence of G9a, H3K9me3 levels have been found to be reduced at the promoter of many genes (Feldman et al., 2006; Epsztejn-Litman et al., 2008; Wagschal et al., 2008). Collectively, and whatever the implication of G9a in the tri-methylation of H3K9, these data support a tight control of the expression of autophagy-related genes at the histone methylation level. In the future, it would be interesting to determine the functional impact of this tri-methylation of H3K9 (with respect to the di-methylation) on the expression of the studied genes and therefore on autophagy outcome.

The obtained results for the enrichment of H3K4me3 at the studied loci are, at first glance, less intuitive. Indeed, H3K4me3 is commonly referred to as an ‘activating’ histone modification, as there is strong evidence of H3K4me3 at the transcriptional start site of active genes (reviewed in Howe et al., 2017). However, a significant reduction in the level of trimethylated H3K4 in parallel with the deacetylation of H4K16 was also recently reported for mammalian and yeast cells, in which autophagy was induced with various stimuli (Füllgrabe et al., 2013). Such a fall in the permissive H3K4me3 and H4K16ac during autophagy has been associated with the establishment of a negative feedback regulatory loop to prevent overstimulation of autophagic flux, which could lead to cell death (Füllgrabe et al., 2013, 2014b). Overall, these results highlight the extreme complexity of the mechanisms involved in the control of autophagy, where antagonistic effects act to ensure the best response to environmental stresses.

As for H3K27me3, its stable enrichment at the monitored genes whatever the condition suggested that this epigenetic mark does not play a role in their expression. However, previous findings in *Drosophila* demonstrated that the lysine demethylase dUTX is recruited to autophagy gene promoters and that its knockdown results in increased H3K27me3 (Denton et al., 2013). Whether this discrepancy between our results and this previous study is due to divergences in the regulatory mechanisms of autophagy among species, differences in the experimental design (environmental factors affecting this epigenetic modification), or more simply to a lack of H3K27me3 enrichment at the monitored regions, is worth investigating.

Finally, it should be noted that while the expression of *atg4b*, *lc3b*, and *p62* decreased at 48 h relative to 24 h, the enrichment in H3K9me3 and H3K4me3 on the related loci was not restored. Such a discrepancy between mRNA expression and histone methylation may be the result of a delay between histone methylation modification and any consequential gene expression effects. However, we cannot rule out the possibility that other post-translational modifications to exposed histone amino acid residues (acetylation, phosphorylation, mono-/di-/tri-methylation) as well as other processes (involving DNA methylation or transcription factors) may be at play in the control of the expression of these genes. Undoubtedly, there is still a need to further understand the role histone modifications play in regulating autophagy. The zebrafish is a powerful model organism for studying numerous biological processes, and our recent characterization of an *in vitro* primary culture system for studying adult skeletal muscle stem cell proliferation and differentiation (Froehlich et al., 2013, 2014) and the herein defined starvation protocol offer a powerful system to further to study the regulation of starvation-induced autophagy. Here, for the first time we profiled the distribution of H3K27me3, H3K9me3, and H3K4me3 along *lc3b*, *atg4b* and *p62/sqstm1* loci in starved zebrafish myocytes and link these modifications with the levels of related transcripts. While these data are the first to describe potential epigenetic mechanisms of autophagy regulation in zebrafish, they do warrant further investigations of teleost fasting-related physiology, both at the cellular and organismal levels. Because the zebrafish is widely used in comparative biology, a better understanding of how zebrafish regulate metabolic biology will provide valuable insight into the overall regulation of muscle hypertrophy, autophagy, and/or atrophy.

## MATERIALS AND METHODS

### Primary myoblast isolation and culture

Fish used in this study were reared and handled in strict accordance with the Institutional Animal Care and Use Committee at the University of Alabama at Birmingham. Adult zebrafish (0.45±0.05 g; outbred *Danio rerio*) were obtained from commercial suppliers and maintained in static tanks at 28°C under a 14 h light:10 h dark photoperiod on a commercial diet. Experiments were carried out in accordance with and approved by the University of Alabama at Birmingham Institutional Animal Care and Use Committee. Epaxial muscle from adult zebrafish was isolated and mechanically and enzymatically digested before cells were plated on poly-L-lysine HBr, laminin-coated dishes (1.5-2.0×10^6^ cells per 35 mm dish) as previously described (Froehlich et al., 2014). MPCs were cultured for four days in 10% fetal bovine serum (FBS)/DMEM at 26°C, then three days in 2% FBS/DMEM to facilitate myotube formation. On day 7 of culture, nascent myotubes (Froehlich et al., 2013, 2014) were incubated in a serum- and amino acid-deprived minimum media (MM) for 12, 24, and 48 h. As control mediums, the above minimal medium was supplemented with both essential and nonessential amino acids without 10% FBS (AA medium) or with 10% FBS (AA+S medium) to control for effects of serum withdrawal.

### RNA isolation and quantification

Total RNA was isolated (24 h in AA and AA+S; 12, 24, and 48 h in MM) from cells using RNAzol (Molecular Research Center, Inc., Cincinnati, OH, USA) according to the supplier's protocol. The resultant aqueous supernatants were purified using PureLink^®^ Mini Kit (Life Technologies RNA) and quantified by Nanodrop spectrophotometry (ThermoFisher). Purified RNAs (300 ng) were reverse transcribed using the ImProm-II™ reverse transcription system (Promega) and Oligo-dT primers (Qiagen). Thermocylcing was performed in a Bio-Rad CFX Connect Real-Time System (Roche, Mannheim, Germany) using Quanta PerfeCTa^®^ Sybr^®^ Green SuperMix as previously described (Froehlich et al., 2013). qPCR reactions were performed on 1 µl aliquots of first-strand cDNA samples in a total volume of 10 μl. Gene-specific primers (10 μM; see Table 1) were validated by BLAST confirmation and by dissociation curve (55-95°C over 5 min). No-template control (NTC) reactions were run in tandem to verify primer specificity. Results were quantified using delta-delta Ct following normalization to *β-actin*. Data were analyzed by one-way ANOVA, with Tukey's post hoc test performed as needed. A *P*-value of <0.05 was considered significant.

**Table 1.**
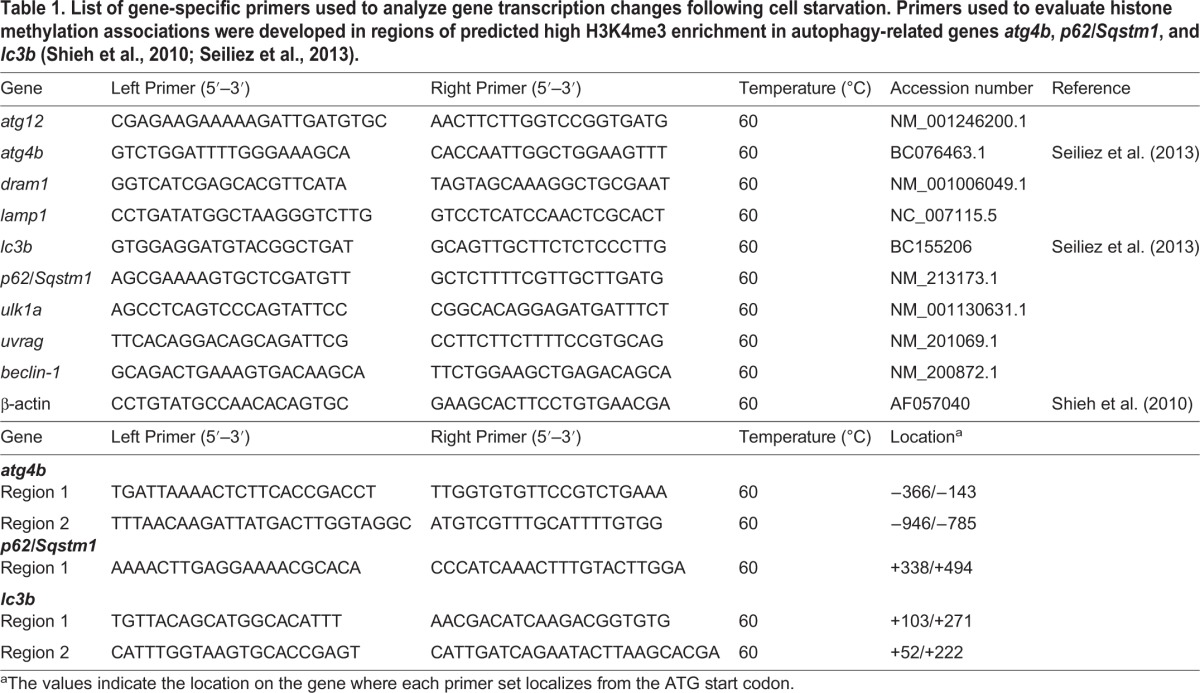
**List of gene-specific primers used to analyze gene transcription changes following cell starvation. Primers used to evaluate histone methylation associations were developed in regions of predicted high H3K4me3 enrichment in autophagy-related genes *atg4b*, *p62/Sqstm1*, and *lc3b* (****Shieh et al., 2010****;** **Seiliez et al., 2013****).**

### Chromatin immunoprecipitation

For chromatin immunoprecipitation (ChIP) experiments, MPCs were cultured as above until day 7 (myotubes) and then fixed following stimulation (24 h in AA+S or 24 and 48 h in MM) for 48 h in 1% methanol-free formaldehyde as previously described (Seiliez et al., 2015). For isolation, three wells were pooled for each ChIP sample (negative/mock ChIP control, total H3, H3K4me3, H3K9me3, and H3K27me3). Prior to ChIP, nuclei were extracted, intact nuclei were resuspended, and DNA was sheared by sonication (13-15 cycles of 15 s pulses with 2 min rests between each pulse) until chromatin was 100-800 bps in size (BioLogics Inc, Cary, NC, USA; Ultrasonic Homogenizer). Nonspecific DNA-agarose bead interactions were blocked by negative control resin incubation (18 h, 4°C). Following column clean-up, diluted (1:2) ChIP reactions were incubated with primary antibodies (negative, normal rabbit IgG; anti-total H3, anti-H3K4me3; anti-H3K9me3; and anti-H3K27me3; Abcam) overnight (∼18 h, 4°C). Antibody-DNA-protein complexes were precipitated on agarose-protein A/G beads, blocked in native/sheared DNA from CHO cells (∼3×10^6^ per 0.65 ml of resin) and BSA (10 mg/ml), and washed (∼18 h, 65°C). DNA was column-purified and samples were analyzed by qPCR in duplicate, as described above, with input (10%) and NTC controls. Primers were designed for autophagy-related genes (*atg4b*, *p62/sqstm1*, and *lC3b*) using the UCSC genome browser (http://genome.ucsc.edu) and the zebrafish ChIP-Seq data from Z-seq (Table 1). Identified primers recognized sites in the upstream regulatory regions with predicted high histone modification enrichment of each target gene. Finally, enrichment was determined by calculating percent input following a correction for the dilution of input (-3.32 cycles). Chromatin immunoprecipitation (ChIP) data were analyzed by Student's *t*-tests between each treatment at each histone mark (H3K4 me3, -K9 me3, -K27me3). Differences were considered significant at *P*<0.05. All statistical analyses and figure generations were completed in GraphPad Prism 7, www.graphpad.com.
